# Primary Succession on a Hawaiian Dryland Chronosequence

**DOI:** 10.1371/journal.pone.0123995

**Published:** 2015-06-12

**Authors:** Kealohanuiopuna M. Kinney, Gregory P. Asner, Susan Cordell, Oliver A. Chadwick, Katherine Heckman, Sara Hotchkiss, Marjeta Jeraj, Ty Kennedy-Bowdoin, David E. Knapp, Erin J. Questad, Jarrod M. Thaxton, Frank Trusdell, James R. Kellner

**Affiliations:** 1 Department of Ecology and Evolutionary Biology, Brown University, Providence, Rhode Island, United States of America; 2 Institute of Pacific Islands Forestry, United States Department of Agriculture Forest Service, Hilo, Hawaii, United States of America; 3 Department of Global Ecology, Carnegie Institution for Science, Stanford, California, United States of America; 4 Department of Geography, University of California Santa Barbara, Santa Barbara, California, United States of America; 5 United States Department of Agriculture, Forest Service, Northern Research Station, Center for Accelerator Mass Spectrometry–Lawrence Livermore National Laboratory, Livermore, California, United States of America; 6 Department of Botany, University of Wisconsin, Madison, Wisconsin, United States of America; 7 Department of Biological Sciences, California Polytechnic University, Pomona, California, United States of America; 8 Department of Biological Sciences, Eastern Kentucky University, Richmond, Kentucky, United States of America; 9 United States Geological Survey, Hawaii Volcano Observatory, Volcano, Hawaii, United States of America; Chinese Academy of Forestry, CHINA

## Abstract

We used measurements from airborne imaging spectroscopy and LiDAR to quantify the biophysical structure and composition of vegetation on a dryland substrate age gradient in Hawaii. Both vertical stature and species composition changed during primary succession, and reveal a progressive increase in vertical stature on younger substrates followed by a collapse on Pleistocene-aged flows. Tall-stature *Metrosideros polymorpha* woodlands dominated on the youngest substrates (hundreds of years), and were replaced by the tall-stature endemic tree species *Myoporum sandwicense* and *Sophora chrysophylla* on intermediate-aged flows (thousands of years). The oldest substrates (tens of thousands of years) were dominated by the short-stature native shrub *Dodonaea viscosa* and endemic grass *Eragrostis atropioides*. We excavated 18 macroscopic charcoal fragments from Pleistocene-aged substrates. Mean radiocarbon age was 2,002 years and ranged from < 200 to 7,730. Genus identities from four fragments indicate that *Osteomeles spp*. or *M*. *polymorpha* once occupied the Pleistocene-aged substrates, but neither of these species is found there today. These findings indicate the existence of fires before humans are known to have occupied the Hawaiian archipelago, and demonstrate that a collapse in vertical stature is prevalent on the oldest substrates. This work contributes to our understanding of prehistoric fires in shaping the trajectory of primary succession in Hawaiian drylands.

## Introduction

Primary succession in forests is characterized by stages of ecosystem development and decline, whereby systems accumulate biomass and vertical stature early in primary succession, but then enter a stage of long-term decline during which biomass and vertical stature are lost. [1,2]. The most typical cause of these changes is persistent phosphorus (P) limitation that occurs with substrate aging [1,3]. Available P increases soon after the formation of parent material due to geochemical weathering, but later becomes limiting and decreases when demand and losses due to leaching outstrip supply [1,3]. However, few studies of primary succession and ecosystem development have been conducted in drylands–those receiving < 500 mm of precipitation annually [4]. Unlike wet systems, dryland ecosystems are likely to increase in vulnerability to fire over the course of primary succession. We know fires have well-known impacts on ecosystems that are associated with successional changes, but our understanding of primary succession in dryland ecosystems is limited, because weathering is slow and the impact of fire across scales of space and time is difficult to quantify [2,4–9].

The Hawaiian archipelago is one of a handful of places in the world where sequences of volcanically derived substrates are of sufficient ages to study primary succession in dryland systems [10]. Clearly defined substrate-age gradients that emanate from a common source in the Hawaiian hotspot enable detailed mapping of substrate ages and associated geochemical conditions. Topography varies largely independently from substrate age and fertility. Climate is controlled by elevation and orientation to the prevailing northeastern trade-winds, and the leeward flanks of the high islands support extensive dryland ecosystems that transcend gradients in temperature, topography and substrate age. Together these features isolate particular processes of interest while holding others constant [3,11,12].

The prevailing mode of disturbance in dryland ecosystems of the Hawaiian Islands today is fire. Conventional views hold that fires are a destructive, anthropogenic introduction and that they do not play a natural role in dictating pathways of primary succession [13]. Microscopic charcoal from dated stratigraphies on the Island of Kauai shows more than an order of magnitude increase in fire associated with the arrival of the first humans to the Hawaiian archipelago [14]. However, during stages of active volcanism, eruption activity is likely to have been a persistent ignition source in the absence of humans [15]. More recent introductions of grazing ungulates and fire-adapted grasses have promoted an ongoing grass/fire cycle in dryland ecosystems that once contained tall-stature dry forest systems [9]. Thus, it is not clear whether fire is a feature of the natural primary succession of these systems, or if it is a recent introduction.

**Fig 1:**
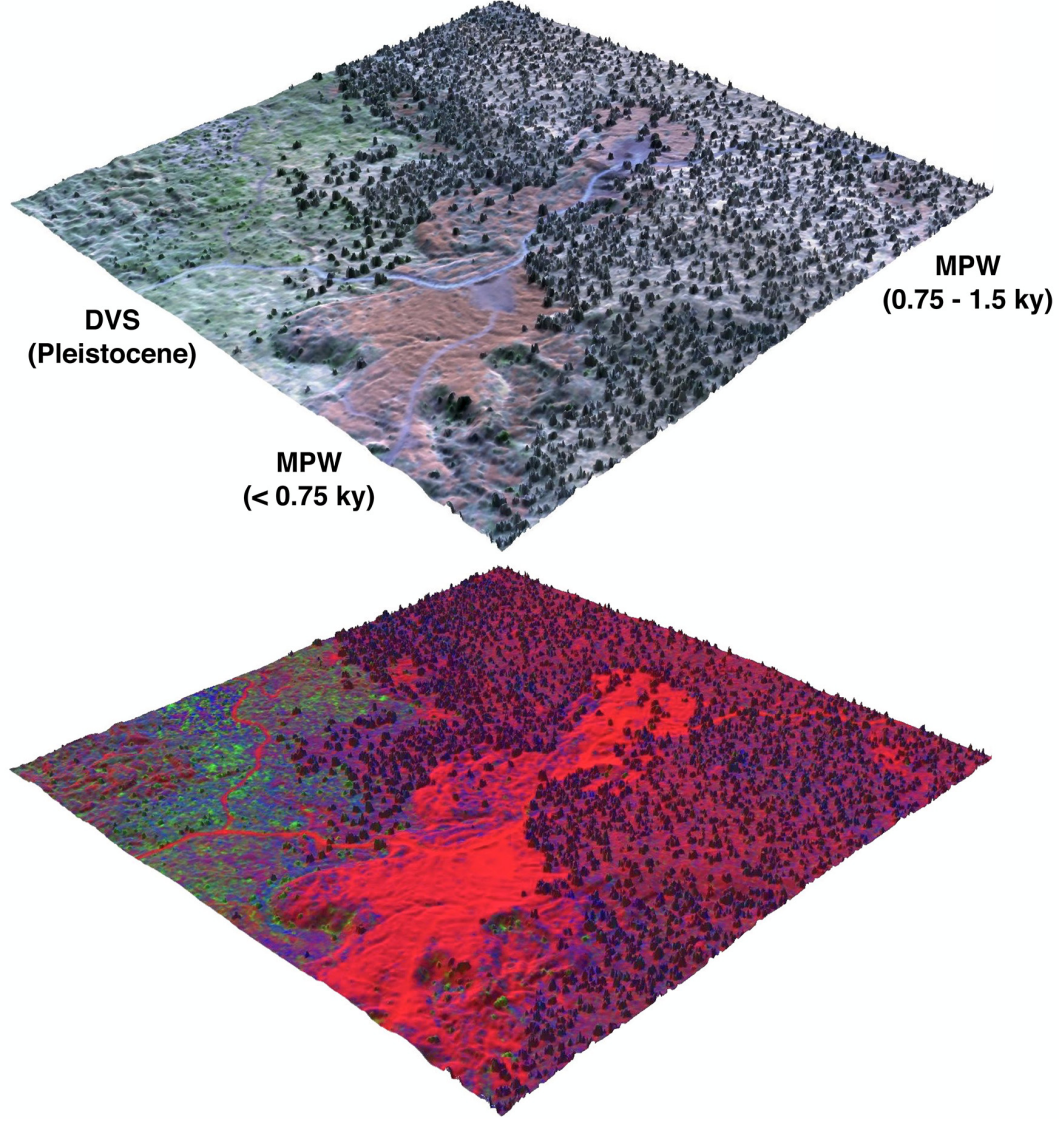
Airborne imaging spectroscopy and LiDAR can be used to quantify the composition and fractional cover of vegetation. The top image is a true color composite overlaid on the digital terrain model (DTM). The bottom image is an RGB composite of the same area processed to quantify barren substrate (B, **r**ed), photosynthetic vegetation (PV; **g**reen) and non-photosynthetic vegetation (NPV; **b**lue). Changes in the lateral distribution of PV, NPV, B, and height are apparent across the substrate age gradient. For example, *M*. *polymorpha* woodland (MPW) on the 750–1200 year-old substrate is dominated by B and tall-statured NPV, indicated by reds and blues in the bottom panel. In contrast, *D*.*viscosa* shrubland (DVS) on a Pleistocene aged substrate is dominated by short-statured NPV and PV, indicated by blues and greens in the bottom panel. The areas shown are a 1 km^2^ sample of the 23.1 km^2^ study area mapped at 2.2 m resolution.

We used measurements from airborne imaging spectroscopy and LiDAR to characterize changes in the structure and composition of vegetation on a volcanic substrate-age gradient on the leeward flanks of Mauna Loa and Mauna Kea on the Island of Hawaii (Fig 1, Table 1, S1 Fig). We show a progressive increase in the vertical stature of vegetation on younger substrates where the lateral distribution of vegetation is sparse followed by a collapse in vertical stature on Pleistocene-aged flows where the lateral distribution of vegetation is dense. We then ask whether histories of fire frequency inferred using radiocarbon dates of macroscopic charcoal excavated from these sites implicate prehistoric and pre-human fires in the pathway of primary succession.

**Table 1:** Site characteristics of the Pōhakuloa substrate age gradient.

Site	Substrate Age (ky)	Mean Rainfall (mm)	Mean Elevation (m)	Lava Type	Hectares
1	0.146	506	1646	Pāhoehoe	477.1
2	0.270	535	1642	Aʻa	152.0
3	0.75–1.5	491	1652	Pāhoehoe	541.9
4	0.75–1.5	471	1644	Aʻa	243.9
5	1.5–3	493	1647	Pāhoehoe	300.3
6	1.5–3	487	1642	Aʻa	93.9
7	3–5	478	1665	Pāhoehoe	50.0
8	3–5	475	1613	Aʻa	2.0
9	10–65	530	1648	Older volcanic rock	452.2
10	10–65	531	1650	Older volcanic rock	452.2

## Material and Methods

Our analysis was conducted on the Pōhakuloa substrate age gradient (PSAG) within the US Army’s Pōhakuloa Training Area (PTA), a 439 km^2^ subalpine dryland ecosystem [16]. This landscape is in the flow path of Mauna Loa, which has erupted 39 times since 1832, and has been active throughout the Holocene [15]. We focus on five substrate ages that span the first 65 ky of ecosystem development between 1600 and 1700 m above sea level (Table 1). These substrates are closely matched in terms of mean annual rainfall and temperature and include both pāhoehoe and aʻa lava types. Pāhoehoe is formed by smooth lava that has a flowing, ropy surface. Aʻa is rough and formed of lava blocks called clinker. These two lava types differ in physical features and in water-holding capacity. Whether magma becomes pāhoehoe or aʻa is determined by its temperature, viscosity and gas content.

### High-resolution surface cover mapping

We used airborne remote sensing to quantify vertical and horizontal structure across the PSAG. The Carnegie Airborne Observatory (CAO) is an airborne remote sensing and analysis system developed to obtain spatially detailed measurements of structural and biochemical properties of vegetation and ecosystems [17]. In this analysis, it combined the airborne visible and infrared imaging spectrometer (AVIRIS) with a LiDAR sensor and 3-D navigation technology (i.e. the CAO Beta system, [17]). We used canopy height measurements from LiDAR to quantify vertical and horizontal vegetation structure, and reflectance observations from the imaging spectrometer to estimate the fractional cover of photosynthetic vegetation (PV), non-photosynthetic vegetation (NPV), and barren volcanic substrate (B) at 2.2 m resolution [18]. Airborne measurements were acquired on 7 January 2008. The LiDAR system was configured to record the locations of up to four reflecting surfaces for every emitted laser pulse at 1.1 m laser spot spacing. Asner *et al*. [17] discuss the positional accuracy of the CAO LiDAR system in detail. Absolute horizontal accuracies of + 0.05 /- 0.08 m (n = 1,684 test points), and vertical accuracies of + 0.06 /- 0.14 m (n = 20,821 test points) were determined from a series of calibration flights over an airport runway at 750–3000 m above ground level [17]. Laser ranges were combined with navigation information to determine the vertical and horizontal locations of reflecting surfaces. We estimated canopy height by generating a raster digital canopy model (DCM), and we processed LiDAR elevation measurements to distinguish laser pulses that were likely to have penetrated vegetation and reached the ground surface. We used these points to interpolate a raster digital terrain model (DTM). The remaining points were used to interpolate a raster digital surface model (DSM). Subtraction of the DTM from the DSM produced the DCM. Subsequent analyses were performed directly on the elevation models.

We quantified the fractional cover of photosynthetic vegetation (PV), non-photosynthetic vegetation (NPV), and bare substrate (B) using spectral mixture analysis applied to imaging spectroscopy data [18]. We compared distributions of height and of fractional cover of PV, NPV, and B on each substrate-age class and lava type using the nonparametric Kolmogorov-Smirnov test [19]. NPV is a metric of cellulose and lignin content in plant tissues and can therefore be used to quantify fire fuels [1,11,12]. We investigated variations in the spatial distribution and magnitude of NPV during primary succession as barren substrates develop into forest and grassland ecosystems. For each substrate age and type, we quantified the lateral distribution NPV by calculating the mean distances from 2000 randomly selected pixel locations to the nearest pixel in each of the following NPV magnitude classes ≥ 25%, 50% or 75%.

### Determining community composition

Previous work in lowland dryland ecosystems in Hawai’i has demonstrated that primary succession follows predictable patterns in species distribution and abundance [20]. We characterized the association between dominant species and substrate ages using a GIS database developed by Shaw and Castillo [16]. Five community types characterized by dominant tree species represent 98.4% of the surface area on PSAG substrates. The remaining 1.6% is other community types. *Metrosideros polymorpha* woodlands (MPW) occur on substrates < 3 ky [16,20]. They are intact native communities, and sometimes support understories with *Dodonaea viscosa* and *Leptecophylla tameiameiae*. *Myoporum-Sophora* dry forest (MSDF) represents short-stature tree communities dominated by the endemic tree species *Myoporum sandwicense* or *Sophora chrysophylla*. MSDF support understories with both native and exotic grass species, including the native *Eragrostis atropoides* and the exotic *Cenchrus setaceus*, and occasional native and exotic shrubs and forbs [21]. *L*. *tameiameiae* shrublands (STY) occur on intermediate aged substrates with minimal soil development. *D*. *viscosa* shrublands (DVS) are on Pleistocene-aged Mauna Kea flows that include the native grass *Eragrostis atropoides* in addition to other exotic grasses and forbs and the native shrub *D*. *viscosa*. Ages of Pleistocene flows in this area are poorly constrained, but have been reported between 10 and 65 ky [22]. We therefore refer to this substrate as 65 ky.

### Quantifying charcoal frequency

We quantified the frequency of charcoal in soils as an index of historical fire frequency within five soil pits that we excavated to a depth of ≤ 1.5 m. We used the DTM and maps of vegetation cover from airborne imaging spectroscopy to distinguish topographic features that were conducive to stable soil accumulation. In general, areas of descending local topography (i.e. bowls) supported soil accumulation > 1 m in depth (K. Kinney, personal observation). We selected sites for excavation from a pool of suitable sites that were accessible and well-distributed on the 65 ky substrate. At each site we established a soil pit that was 1 m in width (X axis) and 2 m in length (Y axis). We attempted to excavate each soil pit to a depth of 1.5 m below the soil surface (Z axis), but ceased excavation if it was impossible to dig further (e.g., beyond large buried boulders). Within each soil pit we extracted three soil profiles that were 10 and 5 cm along the X and Y axes of the soil pit, respectively. The Z axis of each sample taken from the soil profiles was determined by genetic soil horizon or 5 cm, whichever was less. We exhaustively searched profile samples for macroscopic charcoal fragments by visual inspection using brushes, tweezers and a coarse sieve to sort soil samples in the field. We recorded the X, Y and Z position of each charcoal fragment to the nearest mm. Charcoal fragments were placed in sealed Ziploc bags in the field and stored until they were selected for radiocarbon dating.

### Radiocarbon dating

We determined radiocarbon ages (RCA; ^14^C years before present) for 13 macroscopic charcoal samples using ^14^C dating methods. We removed macroscopic charcoal fragments from stored soil samples using a dissecting microscope and forceps. After collection, we sent samples that were ≥ 1 mg to the Carbon, Water & Soils Research Lab in Houghton, Michigan for pre-treatment and graphitization. Samples that were < 1 mg were sent to Lawrence Livermore National Laboratory for pre-treatment and graphitization. We placed samples into 13 × 100 mm borosilicate culture tubes that were heated at 400 C for two hours. Culture tubes containing samples were inserted into a heating block at 95 C. We then treated samples with 1N HCl for 20 minutes, and then with successive washes of 1N NaOH every 25 minutes until the supernatant solution was clear. Samples were treated with 1N HCl, washed twice with de-ionized water, and dried. We applied water rinses at room temperature to all samples for 1–2 minutes. Samples were dried overnight and weighed and then stored in sealed enclosures.

We graphitized macroscopic soil samples in preparation for ^14^C measurement. Samples were combusted at 900 C for six hours with CuO and Ag in sealed quartz test tubes to form CO_2_ gas. The CO_2_ was reduced to graphite by heating at 570 C in the presence of H_2_ gas and an Fe catalyst [23]. We measured the radiocarbon content of each graphitized sample in autumn, 2012 at the Center for Accelerator Mass Spectrometry, Lawrence Livermore National Lab [24]. We normalized sample measurements to the absolute activity of Oxalic Acid I, the international carbon standard. We report radiocarbon abundance as the modern fraction (Fm) and standard radiocarbon age (RCA). We calculated RCA values using the Libby half-life of 5,568 [25]. Reported radiocarbon values include a background subtraction determined from ^14^C-free coal and a δ^13^C correction to account for isotopic fractionation [25]. We scaled all backgrounds to sample size. The δ^13^C values used for radiocarbon abundance corrections were estimated using the same charcoal samples charcoal samples from which ^14^C dates were acquired. Calibrated dates and/or date ranges for each sample were determined from F^14^C values. Calibrated dates for older samples (F^14^C < 1) were determined using the IntCal calibration curve (Reimer *et al*., 2009) and the software program OxCal 4.1.7 (Bronk, 2009). Calibrated dates for modern samples (F^14^C > 1) were determined using the software program CALIBomb (Reimer *et al*. 2004), and the NH_zone2 (northern hemisphere zone 2) calibration curve of Hua and Barbetti (2004). Reported date ranges are 95% confidence intervals, and take into account the analytical error associated with each measurement.

### Taxonomic identification of charcoal

Macroscopic charcoal fragments, ranging in size from a few mm to 1 cm, were identified. Each fragment was manually fractured in cross, radial and tangential section, and we inspected anatomical features in each of these sections at 40X and 100X magnifications using a microscope with reflective light. Depending on the level of preservation, we identified charcoal fragments at the lowest possible taxonomic level by comparison to charcoal reference collections (Anthropology Department-University of Hawai‘i at Mānoa, Archaeological Research Facility-University of California, Berkeley) and to wood reference collections (Center for Wood Anatomy Research-Forest Products Laboratory, Madison, Wisconsin). We also compared charcoal fragments to photographs of cross, radial and tangential wood sections provided by the Anthropology Department and the Department of Botany-University of Hawai‘i at Mānoa, the Department of Botany-University of Wisconsin-Madison, and the Bishop Museum, Honolulu.

Permission to conduct field study and remote analysis in Pōhakuloa training area was provided by the US Army Garrison, Pōhakuloa, Hawaii and the center for environmental management of military lands at Colorado State University.

## Results

Species composition changed during primary succession. *Metrosideros polymorpha* Woodland (MPW) dominated on the youngest substrates, but was replaced by *Myoporum*-*Sophora* Dry Forest (MSDF) and *Dodonaea viscosa* Shrubland (DVS) on substrates of progressively older ages. Replacement of MPW by MSDF, and of MSDF by DVS occurred earlier in primary succession on pāhoehoe than on aʻa. Substrates < 0.30 ky contained entirely MPW on pāhoehoe and aʻa lava types. Pāhoehoe substrates of 0.75–1.5 ky contained 88% MPW, 11% MSDF, and 1% DVS, whereas aʻa conditions contained entirely MPW. Pāhoehoe substrates of 1.5–3.0 ky were 85% MPW and 15% MSDF, and aʻa substrates were 76% MPW and 24% MSDF. On substrates of 3.0–5.0 ky, pāhoehoe conditions were 60% MPW, 20% STY, 12% MSDF, and 8% DVS, but aʻa conditions were entirely MPW. Pleistocene-aged substrates were 89% DVS, 8% other, and 2% MSDF.

Communities accumulated vegetation height on both pāhoehoe and aʻa substrates < 5 ky, but vegetation height was less on Pleistocene-aged flows (Table 2). Maximum canopy height on the youngest substrates was 13.8 and 15.4 m on aʻa and pāhoehoe respectively. On aʻa substrates, a maximum canopy height of 18.5 m was achieved on substrates of 1.5–3.0 ky. Maximum canopy height on pāhoehoe was 18.1 m on substrates of 0.75–1.5 ky. Maximum canopy height on Pleistocene-aged flows was 6.1 m.

**Table 2:** Distributions of vegetation height on substrates of the Pōhakuloa substrate age gradient.

		Percentage of area within height class (m)									
Substrate age	Substrate type	0–1	1–2	2–3	3–4	4–5	5–6	6–7	7–8	8–9	> 9
< 0.30	Aʻa	99.2	0.4	0.1	0.1	0.1	0.1	0.0	0.0	0.0	0.0
	Pāhoehoe	98.8	0.4	0.3	0.2	0.1	0.1	0.0	0.0	0.0	0.0
0.75–1.5	Aʻa	86.8	2.5	2.3	2.2	2.0	1.5	1.1	0.7	0.4	0.5
	Pāhoehoe	82.2	3.8	3.2	3.0	2.5	1.9	1.4	0.9	0.6	0.6
1.5–3	Aʻa	81.7	2.8	2.5	2.4	2.3	2.0	1.7	1.4	1.1	2.2
	Pāhoehoe	81.6	4.0	3.3	3.0	2.6	2.1	1.4	0.9	0.5	0.5
3–5	Aʻa	91.8	1.9	1.8	1.6	1.0	0.6	0.4	0.2	0.2	0.5
	Pāhoehoe	96.1	1.6	0.8	0.4	0.3	0.2	0.2	0.1	0.1	0.1
Pleistocene	Older volcanic rock	99.7	0.2	0.1	0.0	0.0	0.0	0.0	0.0	0.0	0.0

**Fig 2:**
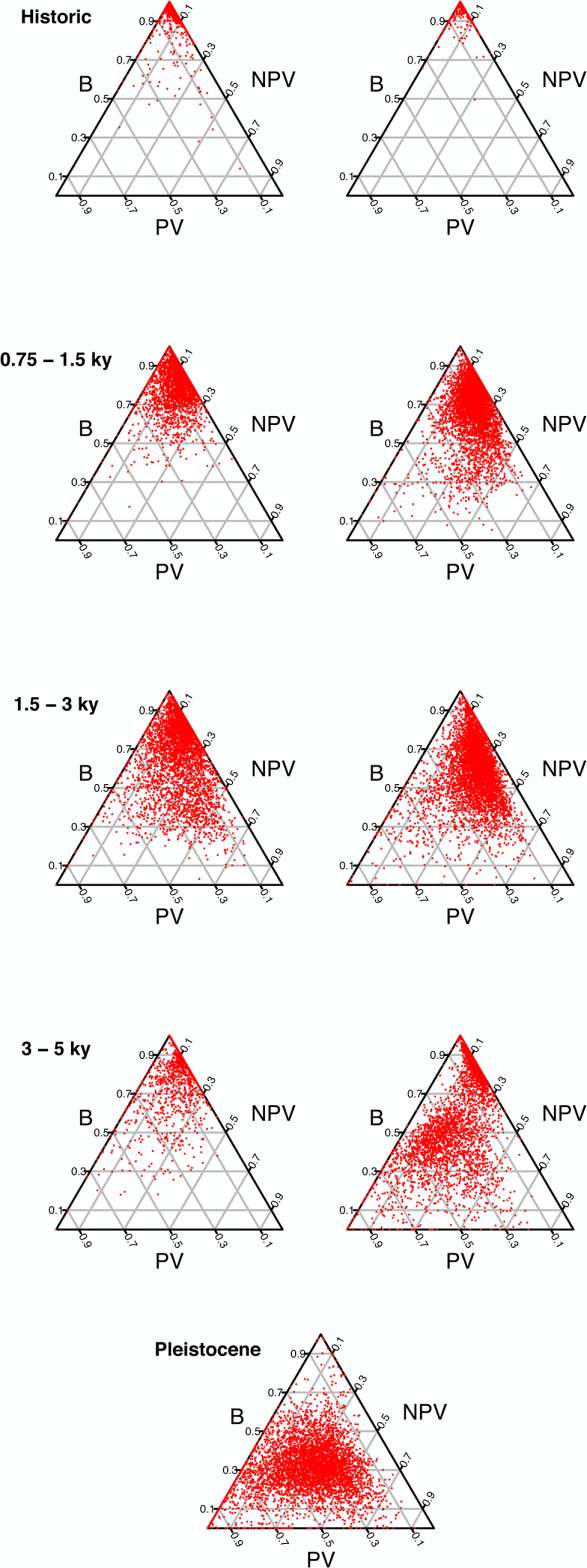
Relationships among types of lateral vegetation cover. PV = photosynthetic vegetation; NPV = non-photosynthetic vegetation; B = barren substrate. The youngest substrates are dominated almost exclusively by B, but accumulate NPV and PV during primary succession and ecosystem development. The rate of development is faster on pāhoehoe (right column) than on aʻa (left column). Pāhoehoe and aʻa lava types are not distinguishable on the 65 ky Pleistocene aged substrates.

The extent and type of lateral vegetation cover changed during primary succession (Fig 2, Table 3). The youngest substrates were dominated by nearly complete barren conditions on pāhoehoe (96.1%) and aʻa (94.1%) respectively (Table 2). Lateral vegetation cover increased on progressively older substrates, and reached a maximum of 69.1% on Pleistocene-aged flows. Both PV and NPV accumulated earlier in primary succession on pāhoehoe substrates than on aʻa (Table 3; K-S test; *P*<0.001).

**Table 3:** Distributions of lateral vegetation cover on substrates of the Pōhakuloa substrate age gradient.

			Percentage of lateral cover									
Substrate age	Substrate type	Vegetation type	0–10	10–20	20–30	30–40	40–50	50–60	60–70	70–80	80–90	90–100
< 0.30	Aʻa	PV	99.0	0.6	0.2	0.1	0.0	0.0	0.0	0.0	0.0	0.0
		NPV	94.7	4.8	0.3	0.1	0.1	0.0	0.0	0.0	0.0	0.0
0.75–1.5	Aʻa	PV	84.7	10.6	3.3	0.9	0.3	0.1	0.1	0.0	0.0	0.0
		NPV	35.5	52.6	10.6	1.1	0.2	0.0	0.0	0.0	0.0	0.0
1.5–3	Aʻa	PV	63.7	18.9	10.1	4.6	1.7	0.6	0.2	0.1	0.0	0.0
		NPV	19.3	44.3	19.4	9.2	5.3	2.0	0.5	0.1	0.0	0.0
3–5	Aʻa	PV	83.1	7.3	4.6	2.5	1.3	0.6	0.4	0.1	0.0	0.1
		NPV	31.3	57.0	8.4	2.5	0.6	0.1	0.0	0.0	0.0	0.0
< 0.30	Pāhoehoe	PV	99.5	0.4	0.1	0.0	0.0	0.0	0.0	0.0	0.0	0.0
		NPV	98.1	1.8	0.1	0.0	0.0	0.0	0.0	0.0	0.0	0.0
0.75–1.5	Pāhoehoe	PV	61.3	25.9	8.2	2.7	1.1	0.5	0.2	0.1	0.0	0.0
		NPV	10.6	43.1	28.6	11.6	4.9	1.0	0.1	0.0	0.0	0.0
1.5–3	Pāhoehoe	PV	42.3	36.5	12.3	4.3	2.2	1.2	0.6	0.3	0.2	0.1
		NPV	7.7	25.2	32.2	22.0	9.3	2.8	0.7	0.1	0.0	0.0
3–5	Pāhoehoe	PV	56.5	5.8	10.5	11.0	7.8	4.2	2.1	1.1	0.5	0.4
		NPV	23.6	49.7	15.8	6.3	3.0	1.2	0.3	0.1	0.0	0.0
Pleistocene	Older volcanic	PV	1.3	6.8	21.8	26.6	18.4	10.1	5.7	3.7	2.5	3.2
	rock	NPV	16.1	14.6	22.4	23.8	14.4	6.1	2.0	0.5	0.1	0.0

Analyses of the lateral cover of dry vegetation indicate that the mean distance between locations with ≥ 25%, 50% or 75% NPV decreased with substrate age, indicating that dry plant material is more densely packed on older substrates (Table 4). Locations with more lateral cover of NPV were separated by greater distances than locations with less lateral cover of NPV. Differences between pāhoehoe and aʻa substrates depended on substrate age. On the youngest substrates, NPV at all thresholds was separated by greater distances on pāhoehoe than aʻa. In contrast, on substrates of 0.75–1.5 and 1.5–3 ky, NPV at all thresholds was separated by shorter distances on pāhoehoe than on aʻa. On substrates of 3–5 ky NPV values ≥ 25% and 75% were separated by greater distances on pāhoehoe than on aʻa, and NPV values ≥ 50% were separated by shorter distances on pāhoehoe than on aʻa.

**Table 4:** Mean distance (m) 2,000 randomly selected locations on substrates of the Pōhakuloa substrate age gradient and the nearest location with at least 25%, 50% or 75% lateral cover of NPV.

		Mean distance (m)		
Substrate age	Substrate type	25% NPV	50% NPV	75% NPV
< 0.30	Aʻa	124.7 (81.6)	158.8 (82.4)	350.7 (163.2)
	Pāhoehoe	249.5 (145.3)	369.6 (154.4)	603.0 (298.4)
0.75–1.5	Aʻa	15.8 (13)	191.8 (135.3)	473.2 (180.1)
	Pāhoehoe	4.7 (4.6)	44.5 (41.0)	262.9 (170.6)
1.5–3	Aʻa	5.9 (5.1)	36.8 (31)	189.4 (159.7)
	Pāhoehoe	3.5 (3.6)	23.1 (28.1)	176.9 (148.7)
3–5	Aʻa	9.9 (6.9)	50.9 (20.7)	(105.7 (33.0)
	Pāhoehoe	12.0 (13.3)	48.4 (36.2)	214.3 (126.8)
Pleistocene	Older volcanic rock	3.5 (4.0)	16.1 (24.1)	108.0 (151.5)

Standard deviation provided in parentheses. Because NPV in this landscape is mainly fine fuels, these numbers demonstrate that a gradient in susceptibility to fire is associated with substrate age.

**Fig 3:**
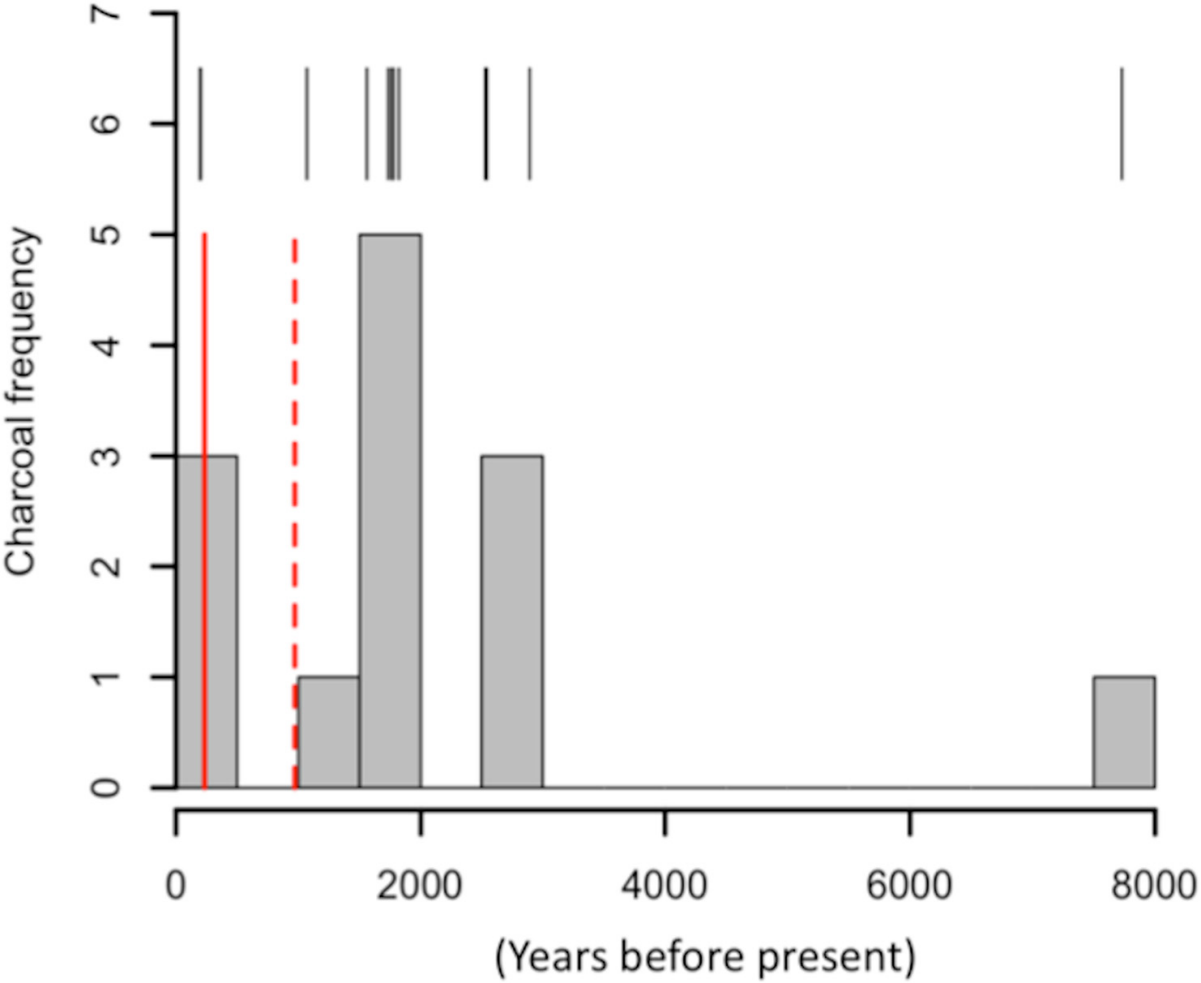
^14^C age frequency distribution for 18 macroscopic charcoal fragments excavated from Pleistocene- aged soils. Black vertical lines indicate charcoal ages. Red vertical lines indicate the timing of arrival of Europeans (solid) and Polynesians (dashed) to the Hawaiian archipelago.(Kirch 2010).

Radiocarbon ages of charcoal fragments ranged from < 200 (2 pieces) to 7,730 (1 piece), with 13 pieces > 1,500 years RCA (Fig 3). We encountered one layer of volcanic tephra from a late-stage Mauna Kea eruption at a depth of 1.2 meters in one soil pit. We obtained genus identities for four macroscopic charcoal fragments. One belonged to *Osteomeles*, one to *Dubautia* and two were identified as either *Metrosideros* or *Psydrax*. These fragments had radiocarbon ages of < 200, 1,775, 2,530, and 7,730 years RCA, respectively. Mean analytical error was ± 0.0033 Fm (i.e. ± 35 yr RCA).

## Discussion

Radiocarbon dating of charcoal collected from 65 ky shrubland indicates that fires have occurred on this landscape for at least 7,380 years RCA (Fig 3). This unambiguously predates the arrival of European and Polynesian settlers to the Hawaiian archipelago [14], and demonstrates that non-anthropogenic fires occurred in Hawaiian drylands prior to human settlement and the introduction of nonnative fire-adapted grasses. Limited evidence suggests that some native species in Hawai’i exhibit characteristics that may be adaptations to fire. For example, the two most common native species on Pleistocene-aged substrates in our study were the native C4 grass *E*. *atropoides*, which accounted for 23.3% of fractional vegetation cover, and the shrub *D*. *viscosa*, which was 12.8% [21]. The shrub *D*. *viscosa* is fire tolerant [13], and quantities of standing dead biomass and leaf moisture in *E*. *atropoides* are similar to the invasive fire-adapted grass *Cenchrus setaceus* (E. Questad, unpublished data). We found little evidence of surface charcoal in MPW on historic substrates, but surface charcoal was abundant in MSDF and DVS on Pleistocene-aged soils. This observation was also made by Stemmerman and Ihle [20] and may indirectly indicate the variation in fire frequency and risk on this substrate-age gradient.

The dominance and eventual replacement of *M*. *polymorpha* on young substrates is relatively well understood. *M*. *polymorpha* is believed to be a poor competitor for water under dry conditions and may be poorly adapted to recovery after fire [20]. The relatively barren conditions on young substrates where *M*. *polymorpha* dominates preempt the establishment and spread of fires, but substrates of intermediate age that support extensive dry vegetation may carry fires that remove *M*. *polymorpha* colonists [20]. On older substrates, our understanding of processes responsible for the transition from dry forests dominated by *M*. *sandwicense* and *S*. *chrysophylla* (MSDF) to short-stature communities dominated by *D*. *viscosa* and native grasses (DVS) is more limited.

Data from airborne imaging spectroscopy indicate that locations with substantial coverage of dry plant material are more densely packed on older substrates (Table 4). Furthermore, this landscape is directly in the flow path of Mauna Loa, which is one of the most active volcanoes on earth. Mauna Loa has erupted 39 times since 1832 [15]. Flows < 0.30 ky intersect Pleistocene-aged substrates on the PSAG at least five times, and intersect substrates of 3–5 ky at least three times. Thus, older substrates contain both the ignition sources and fuel conditions to facilitate grass-fueled fires, despite the fact that they are dominated by species that are native to the Hawaiian Islands.

Our data also indicate that the rate of ecosystem development is dependent on substrate topography. Substrate topography was identified by Jenny [26] as one of five state factors that dictate soil formation. Previous studies have suggested that micro topography can influence rates of primary succession and ecosystem development [27]. We found that pāhoehoe substrates accumulated both lateral cover and vertical stature of vegetation more rapidly than aʻa lava types. This interpretation is based on comparing the percentage cover and height of vegetation on pāhoehoe and aʻa substrates of the same age and lava type (Fig 2, Table 3). Pāhoehoe substrates contain relatively smooth, undulating micro-topography in comparison to the rough, broken surface of aʻa. We suspect that one reason why primary succession might proceed more quickly on pāhoehoe substrates is that undulating texture of pāhoehoe allows water to pool, and directs water flow into cracks where plant growth is possible.

Taken together, evidence of prehistoric fires in this landscape indicates one mechanism by which the vertical stature of vegetation on old, dry substrates could be reduced. It is well known that fires can also cause nutrients to become volatilized when heated, whereas others may be transported off-site in ash. To what degree fires may be influencing the ecosystem structure and productivity in this landscape through nutrient losses is beyond the scope of this paper. However, the data presented here demonstrate that the pattern of primary succession on a dryland substrate age gradient in Hawaii could be responding to a long history of fire.

## Supporting Information

S1 Fig Distributions of lateral vegetation cover and vegetation height on pāhoehoe substrates of the Pōhakuloa substrate age gradient. Panel columns from left to right: photosynthetic vegetation (PV), non-photosynthetic vegetation (NPV), vegetation height (VH), plant community composition and study area photographs. Each row in the figure panel represents a substrate age range **(A)** < 0.30 ky, **(B**) 0.75–1.5 ky, **(C)** 1.5–3.0 ky, **(D)** 3.0–5.0 ky **(E)** 14–65 ky. Plant community composition barplot legend as follows: *Metrosideros polymorpha* woodlands (MPW; orange), *Myoporum-Sophora* dry forest (MSDF; blue), *D*. *viscosa* shrublands (DVS; green), *Leptecophylla tameiameiae* shrublands (STY; yellow), (OTHER; dark orange).
(EPS)

S2 Fig Distributions of lateral vegetation cover and vegetation height on aʻa substrates of the Pōhakuloa substrate age gradient. Panel columns from left to right: photosynthetic vegetation (PV), non-photosynthetic vegetation (NPV), vegetation height (VH), and plant community composition. Each row in the figure panel represents a substrate age range **(A)** < 0.30 ky, **(B**) 0.75–1.5 ky, **(C)** 1.5–3.0 ky, **(D)** 3.0–5.0 ky **(E)** 14–65 ky. Plant community composition barplot legend as follows: *Metrosideros polymorpha* woodlands (MPW; orange), *Myoporum-Sophora* dry forest (MSDF; blue), *D*. *viscosa* shrublands (DVS; green), *Leptecophylla tameiameiae* shrublands (STY; yellow), (OTHER; dark orange).
(EPS)
